# A Boost in Mitochondrial Activity Underpins the Cholesterol-Lowering Effect of Annurca Apple Polyphenols on Hepatic Cells

**DOI:** 10.3390/nu11010163

**Published:** 2019-01-14

**Authors:** Eduardo Sommella, Nadia Badolati, Gennaro Riccio, Emanuela Salviati, Sara Bottone, Monica Dentice, Pietro Campiglia, Gian Carlo Tenore, Mariano Stornaiuolo, Ettore Novellino

**Affiliations:** 1 Department of Pharmacy, School of Pharmacy, University of Salerno, Via Giovanni Paolo II 132, I-84084 Fisciano, SA, Italy; esommella@unisa.it (E.S.); esalviati@unisa.it (E.S.); pcampiglia@unisa.it (P.C.); 2 Department of Pharmacy, University of Naples Federico II. Via Montesano 49, 80149 Naples, Italy; badolatin@gmail.com (N.B.); genriccio@gmail.com (G.R.); sara.bottone@unina.it (S.B.); giancarlo.tenore@unina.it (G.C.T.); 3 PhD Program in Drug Discovery and Development, University of Salerno, Via Giovanni Paolo II 132, I-84084 Fisciano, SA, Italy; 4 Department of Clinical Medicine and Surgery, University of Naples Federico II, Via Pansini 5, 80149 Naples, Italy; monica.dentice@unina.it

**Keywords:** nutraceuticals, apple polyphenols, procyanidin B2, anti-oxidants, cholesterol, statins

## Abstract

Reduction in cholesterol blood levels represents one of the therapeutic goals to achieve in order to reduce the occurrence of cardiovascular diseases. Commonly, this goal is attempted by promoting healthy lifestyle behaviors and low-fat diets. Recently, several nutraceuticals have been shown to possess cholesterol-lowering properties and are becoming common over the counter products. Among others, apple polyphenols efficiently lower total cholesterol levels in humans and impact overall lipid metabolism. *Malus Pumila Miller* cv Annurca is an apple native to Southern Italy presenting one of the highest content of procyanidin B2, a dimeric procyanidin. Tested in clinical trials, the oral consumption of an Annurca polyphenolic extract (AAE) exerted a cholesterol-lowering effect similar to the statins Atorvastatin and Simvastatin. Despite AAE activity, the analysis of the molecular mechanism behind its cholesterol-lowering effect is unclear. Using isotope labeling and high-resolution mass spectrometry approaches we here performed a metabolic profiling of in vitro cultured human hepatocytes treated with AAE to reveal its mechanism of action. The results show that AAE acts differently than statins. The extract reprograms hepatic cell metabolism and promotes mitochondrial respiration, lipolysis and fatty acid β-oxidation. Citrate and acetyl-CoA, both necessary for the production of cholesterol, are diverted to the Krebs Cycle by AAE, that, ultimately, lowers cholesterogenesis and fatty acid synthesis.

## 1. Introduction

Cardiovascular diseases (CVDs) kill more than 4 million people in the world each year accounting for approximately 27% and 32% of all deaths among males and females, respectively [1,2]. Despite improvements in the outcome of these diseases, CVDs risk factors, especially diabetes and obesity, are increasing and CVDs remain leading causes of morbidity and mortality. Prevention of CVDs is undisputed and is being attempted by promoting healthy lifestyle behaviors (e.g., low-fat diet, physical activity, smoking cessation) and by controlling lipids blood levels in individuals at moderate or at high risk of CVDs [1,3,4]. In the last decades, more than half of the reduction in CVD mortality has been attributed to reduction in cholesterol and blood pressure levels. 

Statins are the most common drugs recommended for primary prevention of CVD and are widely prescribed in the treatment of hypercholesterolemia [5]. They competitively inhibit Hydroxymethyl Glutaryl Coenzyme A (HMG-CoA) Reductase, a key enzyme in cholesterol biosynthesis. Statins effectively lower serum cholesterol levels and increase Low-Density Lipoprotein Receptors (LDL-Rs) cell surface expression. By uptaking low density lipoprotein cholesterol (LDL-C) from the blood, LDL-Rs decrease plasmatic concentration of cholesterol, apoB-containing lipoproteins and TG-rich particles. 

While statins are widely prescribed, not all the patients respond to them. Moreover, while they are generally well tolerated, several side effects (mostly myopathy and more rarely rhabdomyolysis) are associated with their consumption [6]. Side effects mainly regard new generation statins that, in virtue of their increased lipophilicity and half-lives, deplete from cholesterol tissues other than liver (especially muscles cells), affecting myocytes plasma membrane and the surrounding sarcolemma [7]. Statin discontinuation induces as well severe side effects and often results in worse cardiovascular outcomes [8].

In the past decades, an increasing number of reports have proved that nutraceuticals may be effective for CVD prevention [4,9,10,11,12] and have a significant effect in reducing population mortality [13,14]. Many dietary products have all shown a certain capability to reduce CVD risk. More importantly, some natural micronutrients and non-nutrient components in these foods, such as polyphenols, have been reported to affect cholesterol metabolism [15,16,17,18,19,20,21]. Among these, procyanidin B2, a dimeric procyanidin, exerts favorable biochemical effects against metabolic disorders and atherosclerosis, two leading causes of CVDs [22,23,24]. 

*Malus Pumila* cv Annurca, an apple native to Southern Italy, contains one of the highest content of procyanidin B2 [25]. Recently, we have published the results of three clinical trials. In the first [26], the daily administration of two Annurca apples led to a reduction of total and LDL-C levels by 8.3% and 14.5%, respectively, and an increase in high density lipoprotein (HDL) levels by 15.2% in healthy subjects. In a second study, 800 mg/day of Annurca apple polyphenolic extract (AAE) substantially impacted both LDL-C and HDL-C (about, −37.6% and +49.3%, respectively), decreasing total cholesterol by about 24.9%. The LDL-C-lowering exerted by AAE was equivalent to the consumption of 10 mg of Atorvastatin or 40 mg of Simvastatin, two widely prescribed statins [27]. In the third one, AAE was shown to increase fecal cholesterol excretion, further confirming their eligibility as a novel complementary safe remedy for CVDs prevention [28].

The safety profile of apple polyphenols has been largely studied on both mice and humans, and, below the 1000 mg/day, no significant hematological, clinical, chemical, histopathological, or urinary effect has been found (Commission Regulation (EC) No. 258/1997). These have made apple containing nutraceuticals popular anti-cholesterol over the counter products. However, despite the many meta-analyses describing their positive effects, data concerning their molecular mechanism of action are far from being complete. Cholesterol lowering activity of polyphenols have been explained (i) by a statin-like mechanism, via inhibition of either HMG-CoA reductase, or squalene synthase (another key enzyme in cholesterol biosynthesis) or (ii) by a β-cyclodextrin-like mechanism via sequestering of lipids and avoidance of their intestinal uptake [28,29,30]. 

Identifying the molecular mechanism of nutraceuticals is notoriously difficult. The polyphenolic fraction of Annurca, for example, is a mixture of hundreds of different metabolites [31,32]. The attempt to identify the molecular mechanism of AAE by testing, individually, each of its components can be misleading, since these components, used as pure molecules, usually show a reduced or even opposite activity compared to the entire phytocomplex. The activity of AAE seems to be more likely the result of a synergism between its components, all influencing each other chemistry, biology and pharmacology [33,34,35].

Here, we make use of Deuterium labeling [36,37], gas chromatography-mass spectrometry (GC/MS) and Fourier transform-ion cyclotron resonance mass spectrometry (FT-ICR) to highlight primary metabolic reactions influenced by AAE in in vitro cultured human hepatocytes. The scenario depicted by our results enlightens unprecedented aspects of AAE cholesterol-lowering activity. AAE acts differently from statins. By promoting mitochondrial OXPHOS, AAE reprograms fatty acid (FA) metabolism in hepatocytes, inhibiting lipogenesis and cholesterogenesis. AAE thus spares acetyl-CoA from becoming HMG-CoA and, instead, diverts it to the Krebs cycle to produce ATP and energy for the cell. 

## 2. Materials and Methods 

### 2.1. Reagents

Chemicals and reagents used for metabolite extraction were all HPLC grade. Water was treated in a Milli-Q water purification system (Millipore, Bedford, MA, USA) before use. The standards used for the identification of intracellular metabolites were from Sigma Chemical Co. (St. Louis, MO, USA). MitoTracker Red CMXRos (M7512, Invitrogen, Carlsbad, CA, USA) used for staining of mitochondria was reconstituted in DMSO and 1mM stock aliquots were stored at −20 °C before use. AAE is a non-standardized industrial procyanidinic extract of Annurca apple PGI ((Protected Geographical Indication) (*Malus Pumila Miller* cv Annurca) produced by MB-Med (Turin, Italy)) [33]. Polyphenols contained in AAE (expressed as μg/mg of dried weight ± s.d.): chlorogenic acid 0.04 ± 0.001; (+) catechin 0.3 ± 0.02; (−) epicatechin 0.3 ± 0.02; procyanidin B_1_ 0.2 ± 0.01; procyanidin trimer 0.3 ± 0.01; procyanidin B_2_ 0.04 ± 0.001; procyanidin trimer (isomer) 0.2 ± 0.02; rutin 0.06 ± 0.002; phloretin-2-*O*-xyloglucoside 0.06 ± 0.003; phloridzin 0.06 ± 0.002. 

### 2.2. Cell Culture and D_2_O Labeling 

HuH7, human hepatoma cells 7 clone 5 (passage 49), were obtained from Ceinge (Naples, Italy) These cells have shown to posses phenotypical stability and can be kept in culture for long-time without accumulating epigenetic changes or losing their differentiated state and function (production of plasma proteins). Cells were cultured (till passage 80) in Dulbecco Modified Eagle Medium (DMEM) (41965-039, GIBCO, Thermo Fisher Scientific, Waltham, MA, USA) supplemented with 10% FBS (10270, GIBCO), glutamine (35050-061, GIBCO), penicillin and streptomycin (15070-063, GIBCO) and sodium selenite (10 μM) in a cell culture incubator at 37 °C and with 5% CO_2_. For D_2_O labeling [36,37], 2 × 10^6^ HuH7 cells were cultivated in a medium supplemented with 5% D_2_O (Sigma Aldrich(St. Louis, MO, USA).When indicated, AAE 400 mg/L, Atorvastatin 10 μM or Simvastatin 10 μM were added to the cultures for 72 h. For in vitro staining of mitochondria with MitoTracker CMX-Ros, a staining solution was prepared by diluting the probe in DMEM to yield a final concentration of 200 nM [38]. Cells were incubated in the presence of the probe for 40 min in a cell incubator at 37 °C and 5% CO_2_. At the end of the incubation, cells were rinsed three times in DMEM, fixed in 4% formaldehyde and visualized under a fluorescent microscope as already described [39]. 

### 2.3. GC-MS

For GC-MS analyses, 2 × 10^6^ HuH7 cells were scraped in ice-cold water and centrifuged at 10,000× *g* for 5 min at 4 °C Membrane pellets were dried and dissolved in 1 mL of ice-cold dichloromethane. Insoluble material was removed by centrifugation at high speed for 10 min at 4° C. The supernatants were dried and resuspended in acetonitrile. Sample was solubilized in pyridine (50μL) and derivatized with 25 μL of N,O-Bis(trimethylsilyl(TMS)trifluoroacetamide (BSTFA) with a reaction time of 90 min. One μL was injected, split ratio 1:10. GC-MS analyses were carried out on a Shimadzu GCMS 2010plus (Kyoto, Japan) with the following parameters. Injection temperature 280 °C, Ramp 0–1.00 min 100 °C, 1.00–6.00 min 100–320 °C, hold for 2.33 min. Column flow 1.10 mL/min Linear velocity 39 cm/s. Helium gas was used. Ion source Temp. 200 °C, Interface 320 °C, Solvent cut 5.9 min, Scan 35–600 m/z. Detector voltage 0.1 kV. Separation was performed on an Agilent (Santa Clara, CA, U.S.A.) SIL-8, 30 m × 0.25 mm, 0.25 μm. 

### 2.4. Mass Spectrometry-Based Metabolomic, Statistics, and Analysis.

Upon treatment with AAE, 2 × 10 ^6^ HuH7 cells were rinsed three times in PBS to be then homogenized in 1 mL of pre-chilled methanol/water 1:1 solution containing 10 nmol of internal standard and centrifuged at 10,000× *g* for 10 min at 4 °C [40]. The resulting supernatants were collected and transferred into new Eppendorf tubes and stored at –80 °C. Analyses were performed in direct infusion following a previous protocol [41] employing a Hamilton syringe (250 μL) at a flow rate of 2 μL/min. Data were acquired on a SolariX XR 7T (Bruker Daltonics, Bremen, Germany). The instrument was tuned with a standard solution of sodium trifluoracetate. Mass Spectra were recorded in broadband mode in the range 100–1500 m/z, with an ion accumulation of 20 ms, with 32 scans using 2 million data points (2M). Nebulizing (N_2_) and drying gases (air) were set at 1 and 4 mL/min, respectively, with a drying temperature of 200 °C. Both positive and negative ESI ionizations were employed. Five replicates of each injection were carried out. The instrument was controlled by Bruker FTMS Control, MS spectra were elaborated with Compass Data Analysis version 4.2 (Bruker), identification of compounds based on accurate MS measurements was performed by Compound Crawler version 3.0 and Metaboscape 3.0 (Bruker). Metabolites signals were normalized using internal standards. Comparisons and differences were analyzed for statistical significance by two-way Anova test and Bonferroni post tests analysis. All graphs, bars or lines indicate mean and error bars indicate standard error of the mean (SEM).

### 2.5. Immunofluorescence 

HuH7 cells were seeded on glass coverslips into 24-well cell culture dish at a density of 15,000 cell/well. After twenty-four hours, they were treated for 72 h in the presence of AAE (400 mg/L) or the corresponding volume of vehicle (DMSO). Cells were fixed in 3.7% formaldehyde/PBS (pH 7.4) for 30 min. Formaldehyde was quenched by incubating the cells for 30 min in 0.1 M glycine in PBS. Cells were then permeabilized in 0.1% Triton/PBS, pH 7.4 for 8 min at room temperature (RT) and incubated with a rabbit monoclonal anti LC3B (D11) XP antibody (3868, Cell Signaling Technology, Beverly, MA, USA) diluted 1:200 in PBS and detected with a goat anti-rabbit IgG (H&L), DyLight 594 conjugate, (ImmunoReagents, Raleigh, NC, USA) diluted 1:1000 in PBS, for 2 h and 45 min at RT, respectively. Cell nuclei were stained with DAPI (Sigma) and observed under a fluorescence microscope.

## 3. Results

### 3.1. AAE Inhibits Cholesterogenesis in HuH7 Cells 

Our previously published studies have already shown that AAE reduces total cholesterol levels both in vitro, in hepatic cultured cells, as well as in vivo, in humans affected by mild hypercholesterolemia [29,42]. Our results confirm those published by other groups and presenting similar effects exerted by other polyphenolic extracts [16,17,43,44,45,46,47]. However, whether the cholesterol lowering effect of AAE is due to i) reduced cholesterogenesis, ii) increased cholesterol excretion or iii) increased conversion of cholesterol into bile acids remains elusive. The mechanism of action hypothesized for other polyphenolic extracts cannot be simply extended to AAE, since the mechanism of action of each extract seems to strongly depend on the nature and the amount of polyphenols contained in it. 

**Figure 1:**
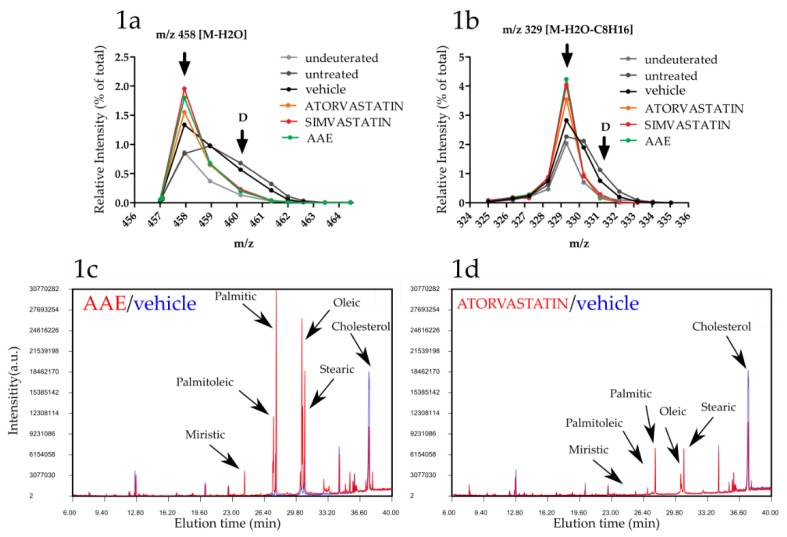
AAE inhibits cholesterogenesis in HuH7 cells. HuH7 cells were grown in the presence of D_2_O and treated for 72 h in the presence of Atorvastatin (Orange dots and lines), Simvastatin (Red), Annurca polyphenolic extract (AAE (green) or the corresponding volume of vehicle (DMSO, black) or water (untreated, dark gray). HuH7 were as well grown in the absence of D_2_O (undeuterated, light gray) or left untreated for comparison. (**a**,**b**) MS analysis of GC peaks eluting at 37.5 min and containing undeuterated TMS derivatized cholesterol (m/z 458 M-H20), a cholesterol fragment (m/z 329 M-H20 -C8H16) and their corresponding deuterated forms (arrows labeled with the letter D). (**c**,**d**) Comparison of gas chromatography-mass spectrometry (GC/MS) spectra (intensity versus retention time) of samples extracted from HuH7 cells treated with AAE (**c**, red profile), vehicle (**c**,**d**, blue profile) or Atorvastatin (**d**, red profile). Arrows indicate cholesterol and fatty acid (FA) over-represented in AAE treated samples. (Representative of at least three experiments).

In order to monitor cholesterogenesis in AAE treated cells we performed D_2_O labeling of in vitro cultured hepatoma cells [36,37]. Briefly, HuH7 cells were grown in a medium supplemented with D_2_O for 72 h, a time sufficient to allow incorporation of Deuterium atoms into de-novo synthesized sterols and fatty acids (FAs). After labeling, lipids were extracted and derivitized with TMS (see methods section for details) to be easily visualized and quantitated by GC-MS. Chemical species endowed with molecular masses heavier (mainly Δm/z of 2–4 Da) than those naturally occurring were found co-eluting with cholesterol and cholesterol fragments (Figure 1a). The presence of these heavier species proves the incorporation of Deuterium in newly synthesized cholesterol and thus that cholesterogenesis was occurring in HuH7 cells. 

When HuH7 cell were cultured in the presence 10 μM Simvastatin or 10 μM Atorvastatin, total intracellular cholesterol levels were reduced (Atorvatatin 0.55 ± 0.09 fold induction compared to vehicle mean ± SEM (standard error of mean), *p* < 0.001; Simvastatin 0.52 ± 0.06, *p* < 0.001) (Figure 1c). Moreover, the intensity of peaks corresponding to deuterated cholesterol molecules were all decreased, confirming, as expected, their molecular mechanism of action resulting in inhibition of de-novo cholesterol synthesis (Figure 1a,b). AAE treatment (400 mg/L) resulted in a reduction of total cholesterol levels (0.48 ± 0.05 fold, *p* < 0.001) with a potency similar to that exerted by the two statins (Figure 1c). AAE reduced as well cholesterol deuterated peaks, proving that its cholesterol lowering activity involves inhibition of de novo cholesterogenesis (Figure 1a,b).

### 3.2. AAE Induces Intracellular Accumulation of FAs

While the effect exerted by Atoravastatin, Simvastatin and AAE on total and newly synthesized cholesterol was similar (Figure 1c,d), some GC peaks were significantly increased in cells treated with AAE (Figure 1c and Figure S1). Among them, we identified the FA palmitic acid (1.78 ± 0.22; *p* < 0.001), palmitoleic acid (2.45 ± 0.20; *p* < 0.001), oleic acid (2.34 ± 0.14; *p* < 0.001), stearic acid (1.17 ± 0.08; *p* < 0.05), and myristic acid (1.73 ± 0.22; *p* < 0.001). In all the other samples (untreated, vehicle and statin treated cells) the intracellular concentrations of the abovementioned FAs were comparable (Figure 1d). MS analysis revealed the presence of deuterated FA species in untreated cells as well as in vehicle treated cells (Figures S2 and S3), indicating that HuH7 cells were engaged in lipogenesis. Differently, no deuterium incorporation could be measured in any of the FAs over-represented in AAE treated cells (Figures S2 and S3), suggesting that the apple polyphenols were not stimulating de novo FA synthesis but, on the contrary, their release from intracellular lipid stores, probably TGs and plasma membrane lipids. 

### 3.3. AAE Reprograms FA Metabolism and Diverts Acetyl-CoA to Krebs Cycle 

**Figure 2:**
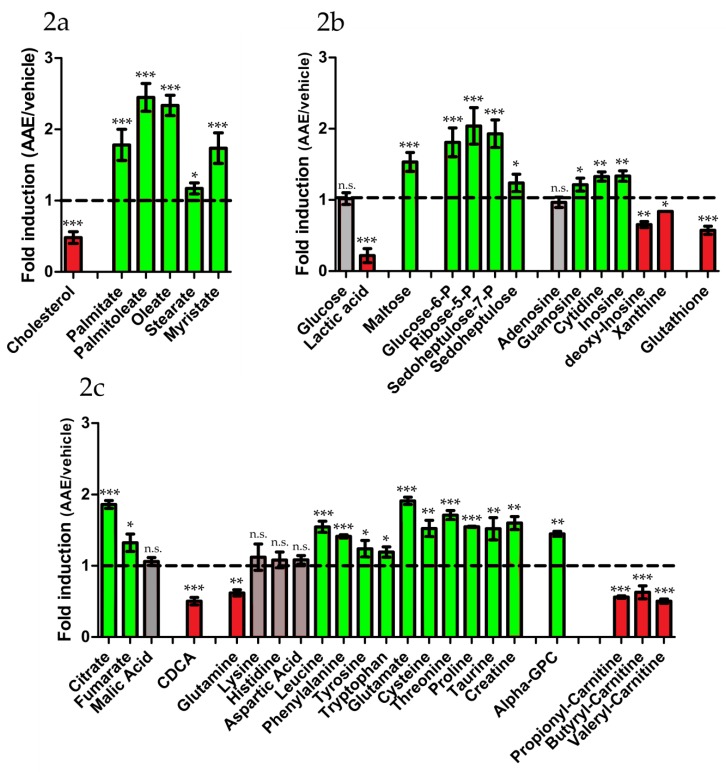
AAE diverts HuH7 metabolism from lactic fermentation toward mitochondrial respiration. Metabolomic profiling of HuH7 cells grown for 72 h in the presence of AAE (400 mg/L). Each bar represents the fold change (AAE versus vehicle) in the intracellular concentration of the indicated metabolites (*n* = 5 measurements, Shown is mean ± SEM Two way ANOVA and Bonferroni post test analysis were performed; *** *p* < 0.001; ** *p* < 0.01; * *p* < 0.05; n.s. non statistically different). Colors are used to highlight increased (green), reduced (red) and unaltered (gray) intracellular metabolites. (CDCA, chenodeoxycholic acid; alpha-GPC, glyceryl phosphoryl choline)).

The effect exerted by AAE on intracellular cholesterol and FA levels (summarized in Figure 2a), prompted us to further extend the metabolic profiling of AAE treated HuH7 cells looking for other metabolites altered by the treatment with the polyphenolic extract (Table 1 and Table S1). Metabolic profiling was performed by Direct Infusion FT-ICR mass spectrometry (DI-FT-ICR-MS), which is characterized by unmatched ultra-high mass accuracy and resolution, that make it highly suitable in metabolite profiling [41]. Metabolomic approaches are extremely useful tools for probing any change in metabolism accompanying drug treatments and provide invaluable insights in the mechanism of action of complex mixtures and phytocomplexes [48]. 

We started our metabolic profiling looking at metabolites that represent energy source for the cell (Figure 2b and Table 1). In virtue of the high rate of their anabolic processes (lipogenesis, cholesterogenesis and protein synthesis) hepatocytes are highly demanding in terms of energy, and use of glucose, amino acids and FAs as energy sources. Intracellular levels of glucose were not altered by AAE (1.02 ± 0.08 fold, *p* > 0.05) while glucose-6-P/fructose 6-P levels (isobaric compounds, 1.81 ± 0.20 fold, *p* < 0.001) were increased by the treatment. Maltose, a by-product of glycogenolysis, was increased by AAE (1.54 ± 0.13 fold, *p* < 0.001) (Figure 2a) pointing toward AAE stimulating conversion of glycogen into glycolysis intermediates. Intracellular levels of lactic acid, the product of pyruvate reduction by Lactate Dehydrogenase, were diminished by AAE (0.22 ± 0.10 fold, *p* < 0.001) (Figure 2c), suggesting that pyruvate in AAE treated cells is mostly transported into mitochondria. 

Pentose phosphate pathway (PPP) is an alternative route toward the glycolysis intermediate glyceraldehyde-3-P and its activity is necessary for reduced glutathione (GSH) and nucleotide production. The intracellular levels of PPP intermediates ribose 5-P (2.04 ± 0.26 fold, *p* < 0.001), sedoheptulose (1.24 ± 0.12 fold, *p* < 0.05), and sedoheptulose 7-P ( 1.93 ± 0.20 fold, *p* < 0.001) were all augmented upon treatment with AAE (Figure 2b). Their accumulation is compatible with an increased rate of PPP activity. Intracellular levels of the nucleotide cytidine (1.33 ± 0.07 fold, *p* < 0.01), guanosine (1.21 ± 0.09 fold, *p* < 0.05), and of inosine (1.34 ± 0.07 fold, *p* < 0.01) were increased by AAE, while adenosine intracellular levels resulted to be not statistically altered by AAE (0.96 ± 0.07 fold, *p* > 0.05) (Figure 2b). Interestingly, GSH levels were reduced upon treatment with AAE (0.57 ± 0.06 fold, *p* < 0.001). 

The first set of results so far described suggests that AAE stimulates glycolysis, PPP (both oxidative and non-oxidative branches of PPP) but not GSH and lactate production. In the absence of lactic fermentation, pyruvate is usually transported into mitochondria and converted in acetyl-CoA and citrate to be used into the Krebs cycle. In our metabolic profiling, citrate levels resulted to be increased in cells treated with AAE (1.86 ± 0.06 fold, *p* < 0.001) (Figure 2c), supporting the hypothesis of an increased transport of pyruvate into mitochondria. In cells like hepatocytes, where lipogenesis and cholesterogenesis occur, citrate is rapidly exported out of the mitochondria and used as substrate to produce malonyl-CoA, necessary for biosynthesis of palmitate and other FAs. Malonyl-CoA is also the precursor of HMG-CoA. Despite the increase in citrate, both FT-ICR and GC-MS profiling (Figure 1 and Figure 2a, Table 1) clearly indicate that cholesterogenesis and lipogenesis are decreased upon treatments with AAE. Moreover the intracellular levels of the bile acid chenodesoxycholic acid (CDCA), that for its synthesis requires cholesterol, is decreased upon treatment with AAE (0.50 ± 0.05 fold, *p* < 0.001) (Figure 2c). This further confirms: i) AAE ability to halt anabolic reactions involved in cholesterogenesis and ii) excludes stimulation of cholesterol conversion into bile acids as a likely mechanism underpinning AAE cholesterol-lowering activity.

Increased levels of citrate can also lead to an increased mitochondrial respiration. In line with this hypothesis, the Krebs cycle intermediate fumarate is also increased by AAE (1.32 ± 0.12 fold, *p* < 0.05).

To prove that the treatment with Annurca polyphenols was indeed increasing mitochondrial activity, we used the mitochondrial probe Mito Tracker CMX-ROS. The fluorescence emitted by this dye correlates with the membrane potential of the mitochondrial inter-membrane space. The latter, depending on the amount of protons transported by the electron transport chain, is a direct measurement of mitochondrial activity. Analysed by fluorimetry, HuH7 cells treated with AAE showed an increased mitochondrial activity compared to cells treated with vehicle (1.22 ± 0.02 fold, *p* < 0.01, Figure S4) confirming that AAE ignites mitochondrial respiration. Differently, AAE did not induce autophagy in HuH7 cells, as shown by the intracellular staining of the autophagy marker LC3B. The number and the size of LC3B positive punctated structures appeared, actually, decreased in AAE treated cells compared to vehicle treated cells (Figure S5).

Pyruvate produced by glycolysis is not the only fuel for mitochondrial activity. Several intracellular metabolites can be involved in metabolic pathway igniting mitochondrial respiratory activity. Glutamine can be converted into glutamate and enter the Krebs cycle as alpha-ketoglutarate. Glutamine levels were reduced by AAE suggesting that glutamine can be indeed one of the sources of increased mitochondrial activity (0.62 ± 0.04 fold, *p* < 0.01). The use of glutamine for catabolic reactions rather than for anabolism would be also indirectly confirmed by its anabolic products GSH, as already shown, reduced by AAE.

Differently, the intracellular levels of other amino acids (both ketogenic and glucogenic ones) are either unaltered or increased by AAE excluding them as possible energy source for mitochondrial activity. Lysine (0.97 ± 0.12 fold, *p* > 0.05), histidine (1.08 ± 0.11 fold, *p* value > 0.05), or aspartate (1.08 ± 0.06 fold, *p* > 0.05) are unaltered by AAE while, on the contrary leucine (1.54 ± 0.08 fold, *p* < 0.001), phenylalanine (1.41 ± 0.02 fold, *p* < 0.001), tyrosine (1.24 ± 0.12 fold, *p* < 0.05), tryptophan (1.19 ± 0.07 fold, *p* < 0.05), glutamate (1.91 ± 0.05, *p* < 0.001), cysteine (1.52 ± 0.11, *p* < 0.01), threonine (1.71 ± 0.07 fold, *p* < 0.001) and proline (1.54 ± 0.01 fold, *p* < 0.001) were all increased by treatment with AAE.

Probably spared from being used as substrate for mitochondrial activity amino acids are likely stored intracellularly for other metabolic reactions (like protein production). In support of this hypothesis, taurine (1.52 ± 0.16 fold, *p* < 0.01) (a derivative of cysteine) and creatine, a derivative of arginine (1.60 ± 0.09 fold, *p* < 0.01), are both increase by AAE.

Mitochondrial and peroxisomal β-oxidation both represent an alternative fuel for hepatic mitochondria. In AAE treated HuH7 cells, we measured a significant decrease in the intracellular level of short chain acyl-carnitines (scfa-carnitines), suggestive of their utilization in the Krebs Cycle. These are produced by peroxisomes via β-oxidation of long chain FA and coupled to carnitine in order to be transported into the mitochondrial matrix and enter the TCA cycle. AAE treatment decreases the intracellular levels of butyril-carnitine (0.56 ± 0.02 fold, *p* < 0.001), propionyl-carnitine (0.54 ± 0.05 fold, *p* < 0.001) and valeryl-carnitine (0.50 ± 0.03 fold, *p* < 0.001) (Figure 2c) all terminal products of peroxisomal FAs catabolism and precursor of TCA cycle intermediate succinate. 

**Figure 3:**
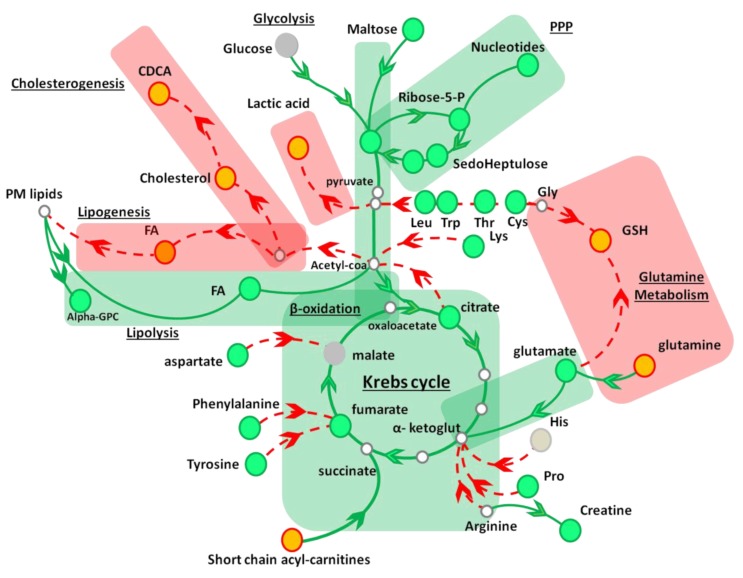
AAE influence on HuH7 metabolome. Schematic cartoon depicting some of the metabolic reactions positively (green boxes) or negatively (red boxes) affected by AAE in HuH7 cells. Red and green arrowheads indicate reactions halted or stimulated by AAE, respectively. Orange and green dots indicate metabolites whose intracellular levels resulted to be decreased or increased by treatment with AAE, respectively. (PPP, phosphate pentose pathway; CDCA, chenodeoxycholic acid; FA, fatty acids; PM lipids; plasma membrane lipids; GSH, reduced glutathione; Leu, leucine; Trp, tryptophan; Thr, threonine; Cys, cysteine; Gly, glycine; Lys, lysine; His, histidine; Pro, proline; alpha-GPC, glyceryl phosphoryl choline).

Overall our metabolite profiling revealed that, in HuH7 cells, AAE stimulates glycolysis and β-oxidation, ultimately increasing mitochondrial respiration (Figure 3 and Table 1). The substrate of β-oxidation seems to be represented by FAs either taken up from the extracellular medium or released by lipolysis from internal stores. Stimulation of membrane lipids hydrolysis by AAE seems to be further confirmed by the increased levels of alpha glyceryl phosphoryl choline (α-GPC; 1.45 ± 0.04 fold, *p* value < 0.01) a byproduct of phospholipase activity. On the contrary, several anabolic reactions occuring in the cytosol (glycogenolysis, lactic fermentation, GSH synthesis) as well as anabolic reactions occurring in the Endoplasmic Reticulum (FA synthesis and cholesterogenesis) were all diminished in vitro by the treatment with AAE. 

Compared to statins, inhibition of de-novo synthesis of cholesterol by AAE is the result of a different mechanism. Statins block selectively HMG-CoA reductase, inhibiting conversion of HMG-CoA into mevalonate, while AAE, on the contrary, modulates the entire metabolic choices of HuH7 halting the usage of citrate for lipogenesis and cholesterogenesis.

## 4. Discussion

While epidemiological evidences suggest that consumption of polyphenols reduces cholesterol levels [4,12], the molecular mechanism behind their cholesterol lowering activity is still under debate. Statins, used for the prevention and treatment of hypercholesterolemia, improves cholesterol levels via inhibition of HMG-CoA reductase a rate-limiting enzyme involved in cholesterol biosynthesis. Several scientific evidences have already suggested that polyphenols may work using a different mechanism. Due to HMG-CoA reductase inactivity, statins upregulate the transcription of *HMG-CoA-R* gene, likely, to counterbalance the overall decreased productivity of its linked pathways [8]. This may also be the reason behind the rapid increase in blood total cholesterol levels measured in patients suddenly interrupting the consumption of statins. None of the reports so far presented have shown that polyphenols induce HMG-CoA Reductase expression, indirectly suggesting a mechanism underpinning their physiological effect different than that used by statins [2]. 

Here, a metabolite profiling of HuH7 cells allowed us to take a snapshot of some of the metabolic pathways modulated by AAE, a nutraceutical enriched in procyanidin B2. The picture we obtained is not yet complete but it is useful to explain some of the cholesterol lowering effects of AAE. Inhibition of de-novo synthesis of cholesterol by AAE is indeed the result of a different mechanism than statins. AAE affects the entire metabolic preferences of HuH7 cells halting the usage of intermediate metabolites for lipogenesis and cholesterogenesis. As a consequence, cells start to obtain FAs from lipid stores, processing TGs present in intracellular lipid droplets and plasma membrane lipids or taken up from the extracellular medium.

Our metabolite profiling (Table 1 and Table S1) revealed that AAE significantly changed the levels of at least 38 key intracellular metabolites in HuH7 cells. The (i) significant elevation of free FAs, (ii) alpha-GPC, (iii) glucose, (iv) the increase in the intracellular level of the PPP intermediates together with (v) the reduction of the intracellular level of glutamine and GSH, all suggest that AAE stimulates glycolysis, lipolysis of membrane lipids, and their β-oxidation. Short chain acyl-carnitines produced by peroxisomal and mitochondrial β-oxidation enter into the Krebs cycle in the form of succinate and further increase mitochondrial respiration, ultimately reducing pyruvate conversion into lactate. By diverting acetyl-CoA and citrate to the Krebs cycle, AAE inhibits anabolic reactions necessary for FAs synthesis and cholesterogenesis.

Previous reports have explained the cholesterol lowering activity of polyphenols with their stimulatory effect on pathways converting cholesterol into bile acids [49]. CYP8B1 and CYP7A1 responsible for catalyzing cholesterol 12α-hydroxylation and 7α-hydroxylation, both steps involved in the conversion of cholesterol into cholic acid (CA) and CDCA [23,24] were both upregulated in Sprague–Dawley strain rats fed a high-fat diet supplemented with apple polyphenols. In our in vitro system CDCA synthesis is decreased in the presence of AAE suggesting that AAE does not stimulate production of primary bile acids. 

The effect of Apple polyphenols have already been correlated to beta-oxidation (mainly peroxisomal) and to regulation of FA metabolism [50]. Mice fed a diet containing apple polyphenols present increased levels of mRNA coding for PPAR-α and PPAR-γ, two genes involved in lipid metabolism [51]. Polyphenols have been shown also to stimulate the expression of acyl-CoA oxidase and dehydrogenase, as well as of Carnitine palmitoyl-transferase and Fatty acyl CoA reductase 1 [52,53,54,55,56,57]. These enzymes catalyze either reactions involved in FA β-oxidation, FA hydrolysis, and transport of lipids into mitochondria for production of acetyl-CoA. In line with these transcriptomic analysis, our results show that AAE increases the intracellular levels of short chain acyl-carnitine butyryl-carnitine, propionyl-carnitine, and valeryl-carnitine all suggestive of AAE stimulating β-oxidation and increasing mitochondrial activity. Moreover, tested as pure molecules, both epicatechin and procyanidin B2, two abundant components of AAE have been shown to possess by themselves uncoupling effects on oxidative phosphorylation in cardiac mitochondria [58], and stimulating fatty acid β-oxidation in cardiac cells. 

## 5. Conclusions

Annurca apples were proved endowed with nutraceutical potential in many human conditions. The hundreds of different metabolites contained in AAE act in synergism [32] and allow this extract to be effective in a plethora of different biological contexts: as antioxidant, as modulator of lipid and cholesterol anabolism, as hair growth promoter [34,35] or against stress and aging [30].

We have recently shown [26,27,28] that the consumption of AAE is able to lower cholesterol levels in humans. The metabolic switch induced by 400 mg/L AAE (an amount corresponding to the daily dosage recommended for consumption in humans) and here described, confirms in vitro the cholesterol lowering activity and enlightens the molecular mechanism behind it, supporting AAE eligibility as candidate nutraceutical against hypercholesterolemia and prevention of CVDs.

## Figures and Tables

**Table 1:** Fold induction for the indicated metabolites measured upon treatment of HuH7 with AAE.

Metabolic Pathway	Metabolite	Fold change ^1^	Metabolic Pathway	Metabolite	Fold Change ^1^
Glycolysis			PPP		
	Glucose	1.02 ± 0.08		Ribose 5-P	2.04 ± 0.26
	Glucose 6-P	1.81 ± 0.2		Sedoheptulose	1.24 ± 0.12
	Lactate	0.22 ± 0.10		Sedoheptulose-7P	1.93 ± 0.20
Glycogenolysis			Nucleotides		
	Maltose	1.56 ± 0.13		Xanthine	0.84 ± 0.01
Amino acids				Adenosine	0,96 ± 0.07
	Proline	1.54 ± 0.04		Cytidine	1.33 ± 0.07
	Threonine	1.71 ± 0.07		Guanosine	1.21 ± 0.09
	Glutamine	0.62 ± 0.04		Inosine	1.34 ± 0.07
	Lysine	0.97 ± 0.12		Deoxy-inosine	0.65 ± 0.04
	Histidine	1.08 ± 0.11		GSH	0.57 ± 0.02
	Cysteine	1.52 ± 0.11	β-oxidation		
	Tryptophan	1.19 ± 0.07		Propionyl-carn	0.54 ± 0.05
	Taurine	1.52 ± 0.16		Butyryl-carn	0.56 ± 0.02
	Creatine	1.60 ± 0.09		Valeryl-carn	0.50 ± 0.03
	Glutamic Acid	1.91 ± 0.05			
	Leucine	1.54 ± 0.08	Krebs cycle		
	Tyrosine	1.24 ± 0.12		Citrate	1.86 ± 0.06
	Phenylalanine	1.41 ± 0.02		Fumarate	1.32 ± 0.12
	Aspartic Acid	1.08 ± 0.06		Malate	1.06 ± 0.05
Lipids			Bile acids		
	Cholesterol	0.48 ± 0.08		CDCA	0.50 ± 0.05
	Palmitic Acid	1.78 ± 0.22			
	Palmitoleic Acid	2.45 ± 0.20			
	Stearic Acid	1.17 ± 0.22			
	Oleic Acid	2.34 ± 0.14			
	Myristic acid	1.73± 0.14			
	α - GPC	1.44 ± 0.04			

^1^ (*n* = 5. Shown is mean ± SEM). Annurca polyphenolic extract (AAE). PPP: pentose phosphate pathway; GSH: glutathione; CDCA: chenodesoxycholic acid.

## Supplementary Materials

The following data are available online at http://www.mdpi.com/2072-6643/11/1/163/s1. Figure S1: GC ion chromatogram of FAs discussed in the manuscript; Figure S2: Mass spectra of FAs discussed in the manuscript; Figure S3: AAE inhibits lipogenesis in HuH7 cells; Figure S4: AAE increases mitochondrial membrane potential of HuH7; Figure S5: AAE does not induce autophagy in HuH7 cells. Table S1: Identification of metabolites in HFs determined by DI- FT-ICR-MS.

## References

[1] Townsend N., Nichols M., Scarborough P., Rayner M. (2015). Cardiovascular disease in Europe--epidemiological update 2015. Eur Hear. J..

[2] Koutsos A., Tuohy K.M., Lovegrove J.A. (2015). Apples and cardiovascular health—Is the gut microbiota a core consideration?. Nutrients.

[3] Catapano A.L., Graham I., De Backer G., Wiklund O., Chapman M.J., Drexel H., Hoes A.W., Jennings C.S., Landmesser U., Pedersen T.R. (2016). 2016 ESC/EAS guidelines for the management of dyslipidaemias. Eur. Heart J..

[4] Shivappa N., Godos J., Hébert J.R., Wirth M.D., Piuri G., Speciani A.F., Grosso G. (2018). Dietary inflammatory index and cardiovascular risk and mortality—A meta-analysis. Nutrients.

[5] Briggs A.D.M., Mizdrak A., Scarborough P. (2013). A statin a day keeps the doctor away: Comparative proverb assessment modelling study. BMJ.

[6] Taylor B.A., Lorson L., White C.M., Thompson P.D. (2015). A randomized trial of coenzyme Q10 in patients with confirmed Statin Myopathy. Atherosclerosis.

[7] Bitzur R., Cohen H., Kamari Y., Harats D. (2013). Intolerance to statins: Mechanisms and management. Diabetes Care.

[8] Thompson P.D., Panza G., Zaleski A., Taylor B. (2016). Statin-associated side effects. J. Am. Coll. Cardiol..

[9] Grosso G. (2018). Effects of polyphenol-rich foods on human health. Nutrients.

[10] Mink P.J., Scrafford C.G., Barraj L.M., Harnack L., Hong C.-P., Nettleton J.A., Jacobs D.R. (2007). Flavonoid intake and cardiovascular disease mortality: A prospective study in postmenopausal women. Am. J. Clin. Nutr..

[11] Hertog M.G.L., Feskens E.J.M., Kromhout D., Hertog M.G.L., Hollman P.C.H., Hertog M.G.L., Katan M.B. (1993). Dietary antioxidant flavonoids and risk of coronary heart disease: The Zutphen Elderly Study. Lancet.

[12] Wang X., Ouyang Y.Y., Liu J., Zhao G. (2014). Systematic review with meta-analysis Flavonoid intake and risk of CVD: A systematic review and meta-analysis of prospective cohort studies. Br. J. Nutr..

[13] Knekt P., Kumpulainen J., Järvinen R., Rissanen H., Heliövaara M., Reunanen A., Hakulinen T., Aromaa A. (2002). Flavonoid intake and risk of chronic diseases. Am. J. Clin. Nutr..

[14] Santini A., Tenore G.C., Novellino E. (2017). Nutraceuticals: A paradigm of proactive medicine. Eur. J. Pharm. Sci..

[15] Tang G.Y., Meng X., Li Y., Zhao C.N., Liu Q., Li H.B. (2017). Effects of Vegetables on Cardiovascular Diseases and Related Mechanisms. Nutrients.

[16] Nagasako-Akazome Y., Kanda T., Ikeda M., Shimasaki H. (2005). Serum Cholesterol-Lowering Effect of Apple Polyphenols in Healthy Subjects. J. Oleo Sci..

[17] Nagasako-akazome Y., Kanda T., Ohtake Y., Shimasaki H., Kobayashi T. (2007). Apple Polyphenols Influence Cholesterol Metabolism in Healthy Subjects with Relatively High Body Mass Index. J. Oleo Sci..

[18] Osada K., Funayama M., Fuchi S., Sami M., Ohta Y., Kanda T., Ikeda M. (2006). Effects of Dietary Procyanidins and Tea Polyphenols on Adipose Tissue Mass and Fatty Acid Metabolism in Rats on a High Fat Diet. J. Oleo Sci..

[19] Opyd P.M., Jurgoński A., Juśkiewicz J., Milala J., Zduńczyk Z., Król B. (2017). Nutritional and health-related effects of a diet containing apple seed meal in rats: The case of amygdalin. Nutrients.

[20] Pirillo A., Catapano A.L. (2015). Berberine, a plant alkaloid with lipid- and glucose-lowering properties: From in vitro evidence to clinical studies. Atherosclerosis.

[21] Zhao C., Meng X., Li Y., Li S., Liu Q., Tang G., Li H. (2017). Fruits for Prevention and Treatment of Cardiovascular Diseases.

[22] Wang S., Melnyk J.P., Tsao R., Marcone M.F. (2011). How natural dietary antioxidants in fruits, vegetables and legumes promote vascular health. Food Res. Int..

[23] Panche A.N., Diwan A.D., Chandra S.R. (2016). Flavonoids: An overview. J. Nutr. Sci..

[24] Serra A.T., Rocha J., Sepodes B., Matias A.A., Feliciano R.P., De Carvalho A., Bronze M.R., Duarte C.M.M., Figueira M.E. (2012). Evaluation of cardiovascular protective effect of different apple varieties— Correlation of response with composition. Food Chem..

[25] Tenore G.C., Campiglia P., Ritieni A., Novellino E. (2013). In vitro bioaccessibility, bioavailability and plasma protein interaction of polyphenols from Annurca apple (M. pumila Miller cv Annurca). Food Chem..

[26] Tenore G.C., Caruso D., Buonomo G., D’Urso E., D’Avino M., Campigli P., Marinelli L., Novellino E. (2016). Annurca (Malus pumilamiller cv. Annurca) apple as a functional food for the contribution to a healthy balance of plasma cholesterol levels: Results of a randomized clinical trial. J. Sci. Food Agric..

[27] Tenore G.C., Caruso D., Buonomo G., D’Avino M., Campiglia P., Marinelli L., Novellino E. (2017). A Healthy Balance of Plasma Cholesterol by a Novel Annurca Apple-Based Nutraceutical Formulation: Results of a Randomized Trial. J. Med. Food.

[28] Tenore G.C., Carotenuto A., Caruso D., Buonomo G., D’Avino M., Brancaccio D., Ciampaglia R., Maisto M., Schisano C., Novellino E. (2018). A nutraceutical formulation based on Annurca apple polyphenolic extract is effective on intestinal cholesterol absorption: A randomised, placebo-controlled, crossover study. PharmaNutrition.

[29] Tenore G.C., Calabrese G., Stiuso P., Ritieni A., Giannetti D., Novellino E. (2014). Effects of Annurca apple polyphenols on lipid metabolism in HepG2 cell lines: A source of nutraceuticals potentially indicated for the metabolic syndrome. Food Res. Int..

[30] Stirpe M., Palermo V., Bianchi M.M., Silvestri R., Falcone C., Tenore G., Novellino E., Mazzoni C. (2017). Annurca apple (M. pumila Miller cv Annurca) extracts act against stress and ageing in S. cerevisiae yeast cells. BMC Complement. Altern. Med..

[31] Sommella E., Ismail O.H., Pagano F., Pepe G., Ostacolo C., Mazzoccanti G., Russo M., Novellino E., Gasparrini F., Campiglia P. (2017). Development of an improved online comprehensive hydrophilic interaction chromatography × reversed-phase ultra-high-pressure liquid chromatography platform for complex multiclass polyphenolic sample analysis. J. Sep. Sci..

[32] Sommella E., Pepe G., Pagano F., Ostacolo C., Tenore G.C., Russo M.T., Novellino E., Manfra M., Campiglia P. (2015). Detailed polyphenolic profiling of Annurca apple (M. pumila Miller cv Annurca) by a combination of RP-UHPLC and HILIC, both hyphenated to IT-TOF mass spectrometry. Food Res. Int..

[33] Riccio G., Maisto M., Bottone S., Badolati N., Rossi G.B., Tenore G.C., Stornaiuolo M., Novellino E. (2017). WNT inhibitory activity of malus pumila miller cv annurca and malus domestica cv limoncella apple extracts on human colon-rectal cells carrying familial adenomatous polyposis mutations. Nutrients.

[34] Badolati N., Sommella E., Riccio G., Salviati E., Heintz D., Bottone S., Di Cicco E., Dentice M., Tenore G., Campiglia P. (2018). Annurca Apple Polyphenols Ignite Keratin Production in Hair Follicles by Inhibiting the Pentose Phosphate Pathway and Amino Acid Oxidation. Nutrients.

[35] Riccio G., Sommella E., Badolati N., Salviati E., Bottone S., Campiglia P., Dentice M., Tenore G., Stornaiuolo M., Novellino E. (2018). Annurca Apple Polyphenols Protect Murine Hair Follicles from Taxane Induced Dystrophy and Hijacks Polyunsaturated Fatty Acid Metabolism toward β-Oxidation. Nutrients.

[36] Esterman A.L., Cohen B.I., Javitt N.B. (1985). Cholesterol metabolism: Use of D2O for determination of synthesis rate in cell culture. J. Lipid Res..

[37] Castro-Perez J., Previs S.F., McLaren D.G., Shah V., Herath K., Bhat G., Johns D.G., Wang S.-P., Mitnaul L., Jensen K. (2011). In vivo D2O labeling to quantify static and dynamic changes in cholesterol and cholesterol esters by high resolution LC/MS. J. Lipid Res..

[38] Johnson S., Rabinovitch P. (2012). Ex vivo imaging of excised tissue using vital dyes and confocal microscopy. Curr. Protoc. Cytom..

[39] Riccio G., Bottone S., La Regina G., Badolati N., Passacantilli S., Rossi G.B., Accardo A., Dentice M., Silvestri R., Novellino E. (2018). A Negative Allosteric Modulator of WNT Receptor Frizzled 4 Switches into an Allosteric Agonist. Biochemistry.

[40] Ser Z., Liu X., Tang N.N., Locasale J.W. (2015). Extraction parameters for metabolomics from cultured cells. Anal. Biochem..

[41] Sommella E., Conte G.M., Salviati E., Pepe G., Bertamino A., Ostacolo C., Sansone F., Prete F.D., Aquino R.P., Campiglia P. (2018). Fast profiling of natural pigments in different spirulina (arthrospira platensis) dietary supplements by DI-FT-ICR and evaluation of their antioxidant potential by pre-column DPPH-UHPLC assay. Molecules.

[42] Tenore G.C., Campiglia P., Stiuso P., Ritieni A., Novellino E. (2013). Nutraceutical potential of polyphenolic fractions from Annurca apple (M. pumila Miller cv Annurca). Food Chem..

[43] Dhaliya S.A., Surya A.S., Dawn V.T., Betty C., Arun K., Sunil C. (2013). A Review of Hyperlipidemia and Medicinal Plants. Int.J.A.PS.BMS.

[44] Agrawal A.D. (2011). Pharmacological activities of flavonoids: A review. Int. J. Pharm. Sci. Nanotechnol..

[45] Amani R. (2014). Flavonoid-rich beverage effects on lipid profile and blood pressure in diabetic patients. World J. Diabetes.

[46] Chavez-Santoscoy R.A., Gutierrez-Uribe J.A., Granados O., Torre-Villalvazo I., Serna-Saldivar S.O., Torres N., Palacios-González B., Tovar A.R. (2014). Flavonoids and saponins extracted from black bean (Phaseolus vulgaris L.) seed coats modulate lipid metabolism and biliary cholesterol secretion in C57BL/6 mice. Br. J. Nutr..

[47] Shao D., Lian Z., Di Y., Zhang L., shahid riaz Rajoka M., Zhang Y., Kong J., Jiang C., Shi J. (2018). Dietary compounds have potential in controlling atherosclerosis by modulating macrophage cholesterol metabolism and inflammation via miRNA. npj Sci. Food.

[48] Want E.J., Masson P., Michopoulos F., Wilson I.D., Theodoridis G., Plumb R.S., Shockcor J., Loftus N., Holmes E., Nicholson J.K. (2013). Global metabolic profiling of animal and human tissues via UPLC MS. Nat. Protoc..

[49] Sunagawa T., Ohta Y., Sami M., Kanda T., Osada K. (2016). Hypocholesterolemic Effect of Dietary Apple Polyphenol Is Associated with Alterations in Hepatic Gene Expression Related to Cholesterol Metabolism in Rats. Int. J. Life Sci. Med. Res..

[50] Masuda I., Koike M., Nakashima S., Mizutani Y., Ozawa Y., Watanabe K., Sawada Y., Sugiyama H., Sugimoto A., Nojiri H. (2018). Apple procyanidins promote mitochondrial biogenesis and proteoglycan biosynthesis in chondrocytes. Sci. Rep..

[51] Abraham Domínguez-Avila J., González-Aguilar G.A., Alvarez-Parrilla E., de la Rosa L.A. Modulation of PPAR expression and activity in response to polyphenolic compounds in high fat diets. Int. J. Mol. Sci..

[52] Ali F., Ismail A., Esa N.M., Pei C.P. (2015). Transcriptomics expression analysis to unveil the molecular mechanisms underlying the cocoa polyphenol treatment in diet-induced obesity rats. Genomics.

[53] Kozłowska A., Szostak-Wegierek D. (2014). Flavonoids—Food sources and health benefits. Rocz. Państwowego Zakładu Hig..

[54] Jung M., Triebel S., Anke T., Richling E., Erkel G. (2009). Influence of apple polyphenols on inflammatory gene expression. Mol. Nutr. Food Res..

[55] Wang N., Liu W., Zhang T., Jiang S., Xu H., Wang Y., Zhang Z., Wang C., Chen X. (2018). Transcriptomic Analysis of Red-Fleshed Apples Reveals the Novel Role of MdWRKY11 in Flavonoid and Anthocyanin Biosynthesis. J. Agric. Food Chem..

[56] Ohta Y., Sami M., Kanda T., Saito K., Osada K., Kato H. (2006). Gene expression analysis of the anti-obesity effect by apple polyphenols in rats fed a high fat diet or a normal diet. J. Oleo Sci..

[57] Shabrova E.V., Tarnopolsky O., Singh A.P., Plutzky J., Vorsa N., Quadro L. (2011). Insights into the molecular mechanisms of the anti-atherogenic actions of flavonoids in normal and obese mice. PLoS ONE.

[58] Kopustinskiene D.M., Savickas A., Vetchý D., Masteikova R., Kasauskas A., Bernatoniene J. (2015). Direct effects of (-)-Epicatechin and procyanidin B2 on the respiration of rat heart mitochondria. Biomed. Res. Int..

