# Supplementary material for: A Boost in Mitochondrial Activity Underpins the Cholesterol-Lowering Effect of Annurca Apple Polyphenols on Hepatic Cells
Source: Nutrients. 2019 Jan 14;11(1):163. doi: 10.3390/nu11010163 (PMC6356966; doi:10.3390/nu11010163)
Supplement: Supplementary file 1 [file nutrients-11-00163-s001.pdf]

Supplementary material for the manuscript

A Boost in Mitochondrial Activity Underpins the Cholesterol-Lowering Effect of Annurca Apple Polyphenols on Hepatic Cells

Eduardo Sommella, Nadia Badolati, Gennaro Riccio, Emanuela Salviati, Sara Bottone, Monica Dentice, Pietro Campiglia , Gian Carlo Tenore, Mariano Stornaiuolo and Ettore Novellino

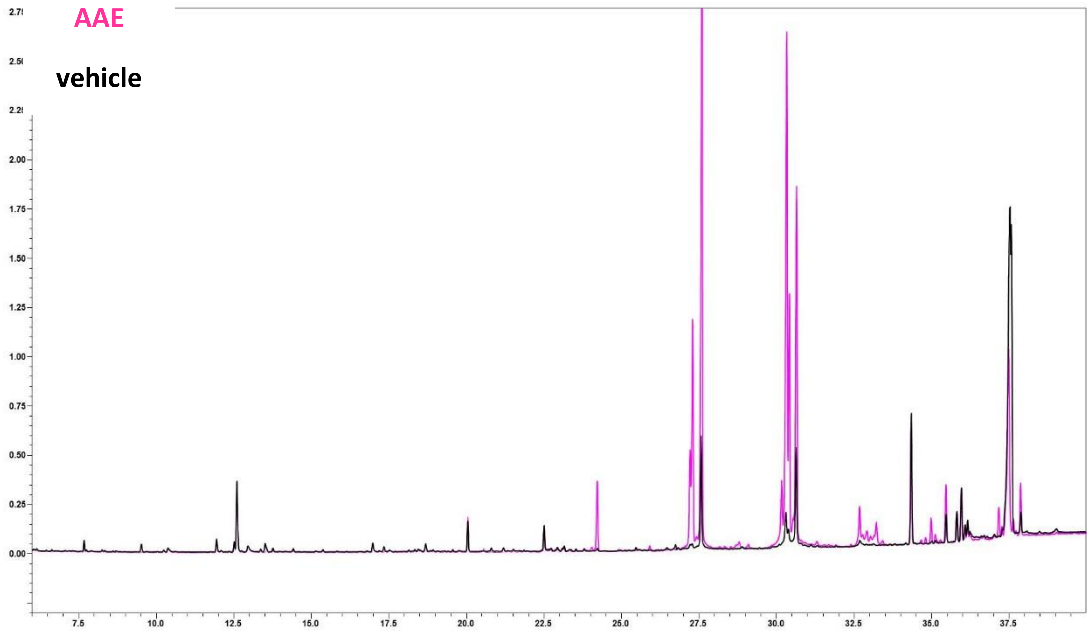

Figure S1. GC-MS total ion chromatogram (AAE (purple) vs Vehicle (black).

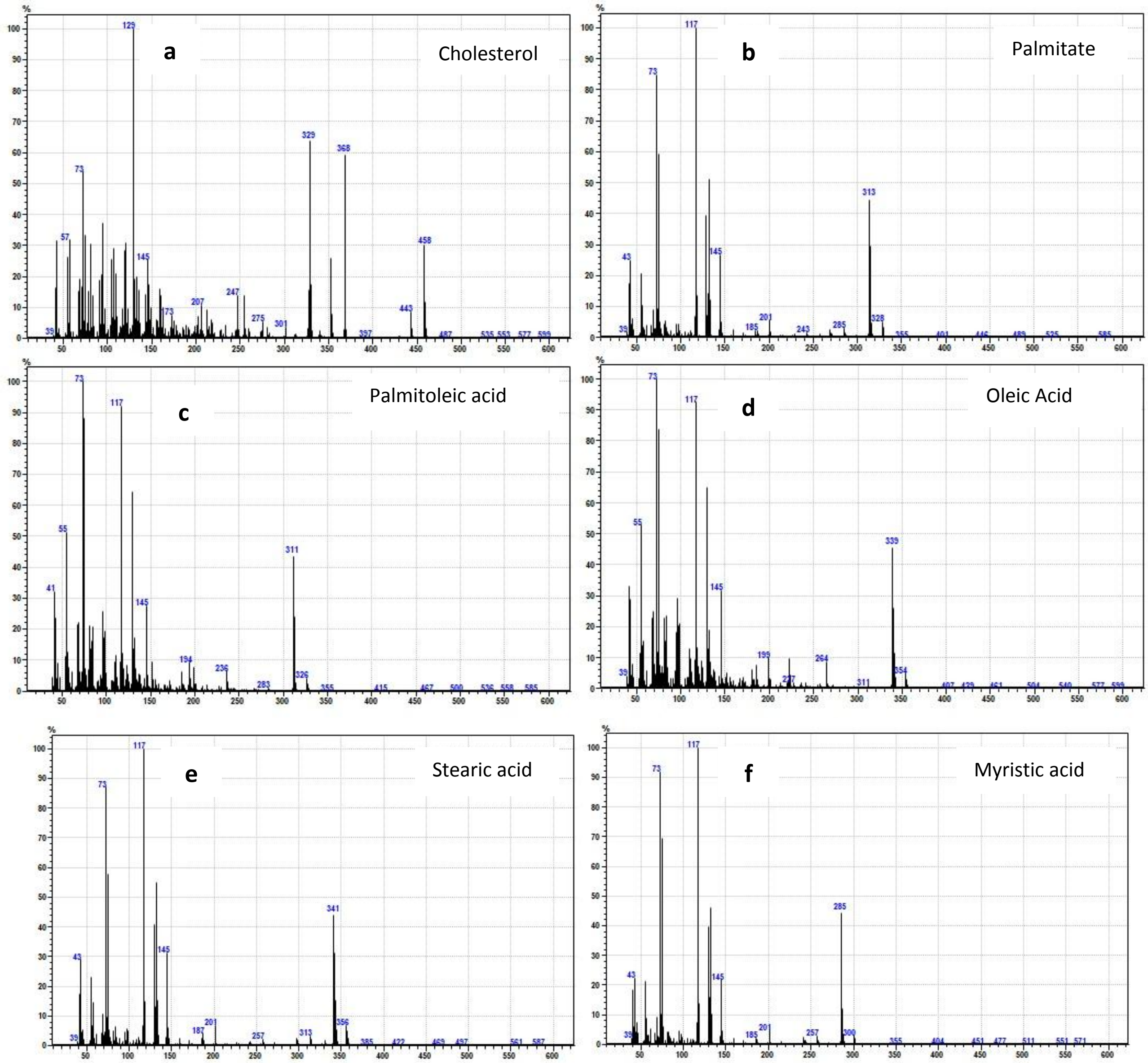

Figure S2: Mass spectra and retention time (RT) of: **a**) cholesterol (RT 37.5 min, (m/z 458, 368, 329)), **b**) palmitate (RT 27.6 min, (m/z 313, 117, 129, 328)), **c**) palmitoleic acid ((RT 27.3 (m/z 311, 326, 129, 117 and 236)), **d**) oleic acid ((RT 30.3 min m/z 354,339,236,129,117)) **e**) stearic acid (RT 30.6, (m/z 356,341,132,117)) and **f**) myristic acid (RT 24.2 (m/z 285,117,132)).

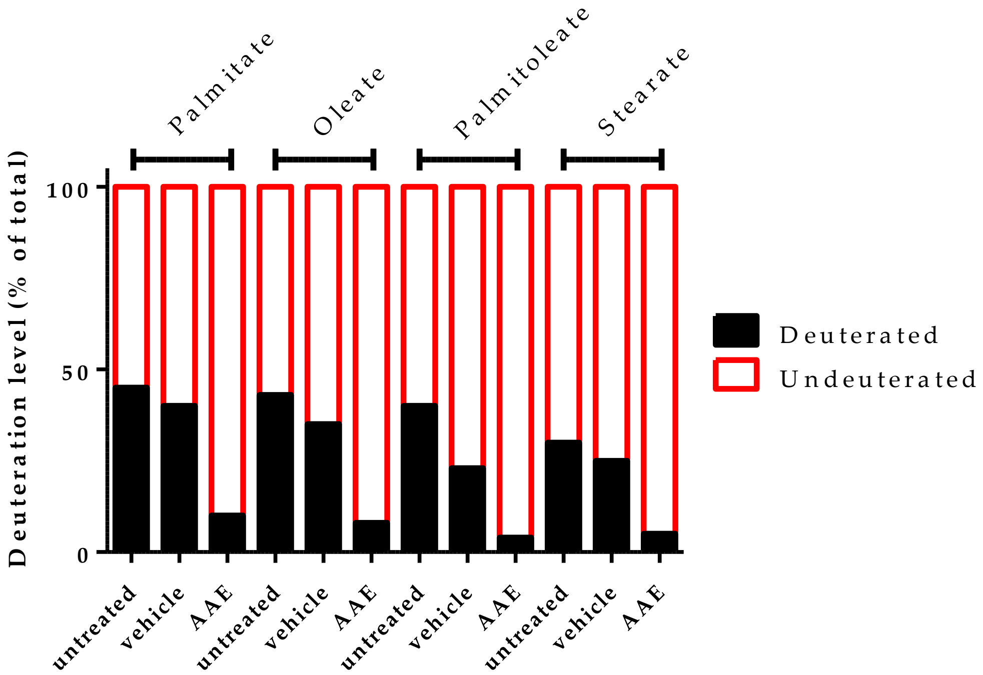

Figure S3. AAE inhibits lipogenesis in HuH7 cells. HuH7 cells were grown in the presence of D_2O and treated for 72 h in the presence 400 mg/L AAE, of the corresponding volume of DMSO (vehicle) or of water (untreated). Deuteration level (relative intensity (% of total)) of chemical species presenting masses heavier than those naturally occurring for the indicated FA species. See Figure 1 for further details.

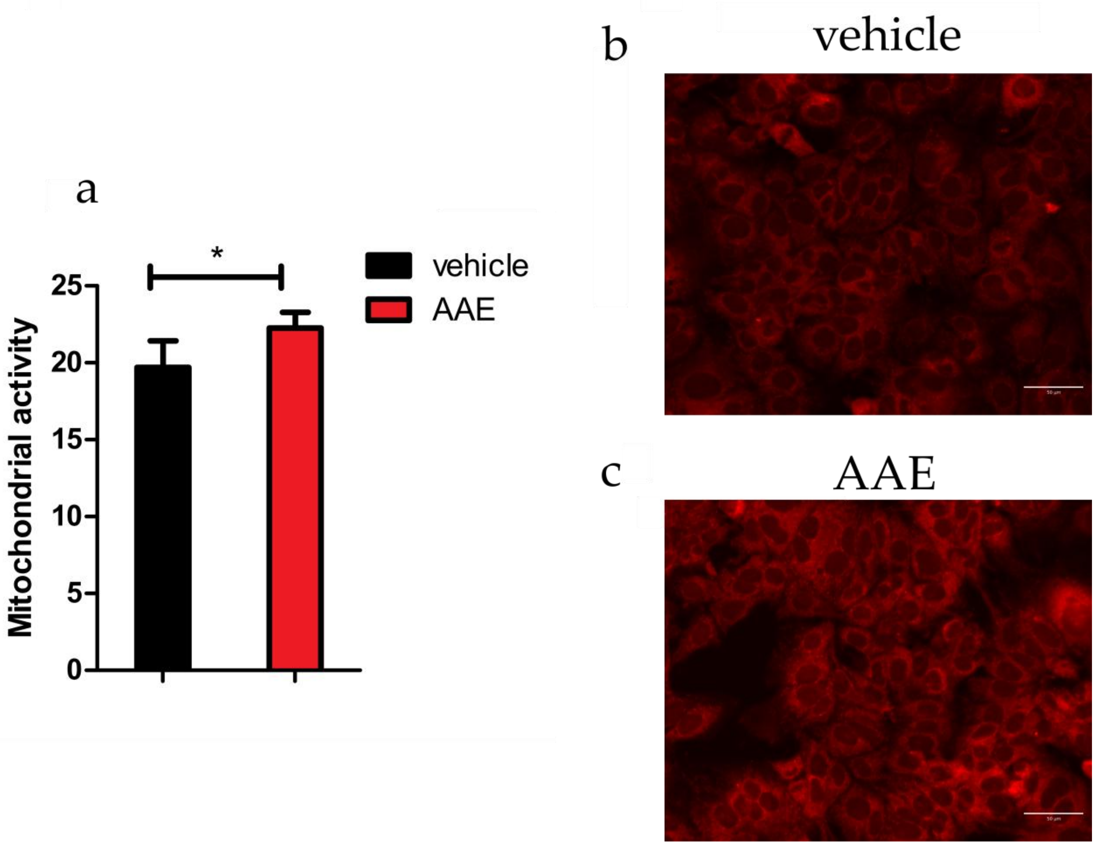

Figure S4: AAE increases mitochondrial membrane potential in HuH7. Cells treated with AAE or with vehicle were incubated with Mitotracker CMX-ROS. a) Quantitation of fluorescence intensity of Mitotracker in HuH7 treated with AAE or with vehicle. * $p < 0.05$. **b-c**) Immunofluorescence analysis showing increased fluorescence emission of Mitotracker CMX-ROS in HuH7 cells treated with AAE. Scale bars in **b** and **c** correspond to 50 μm

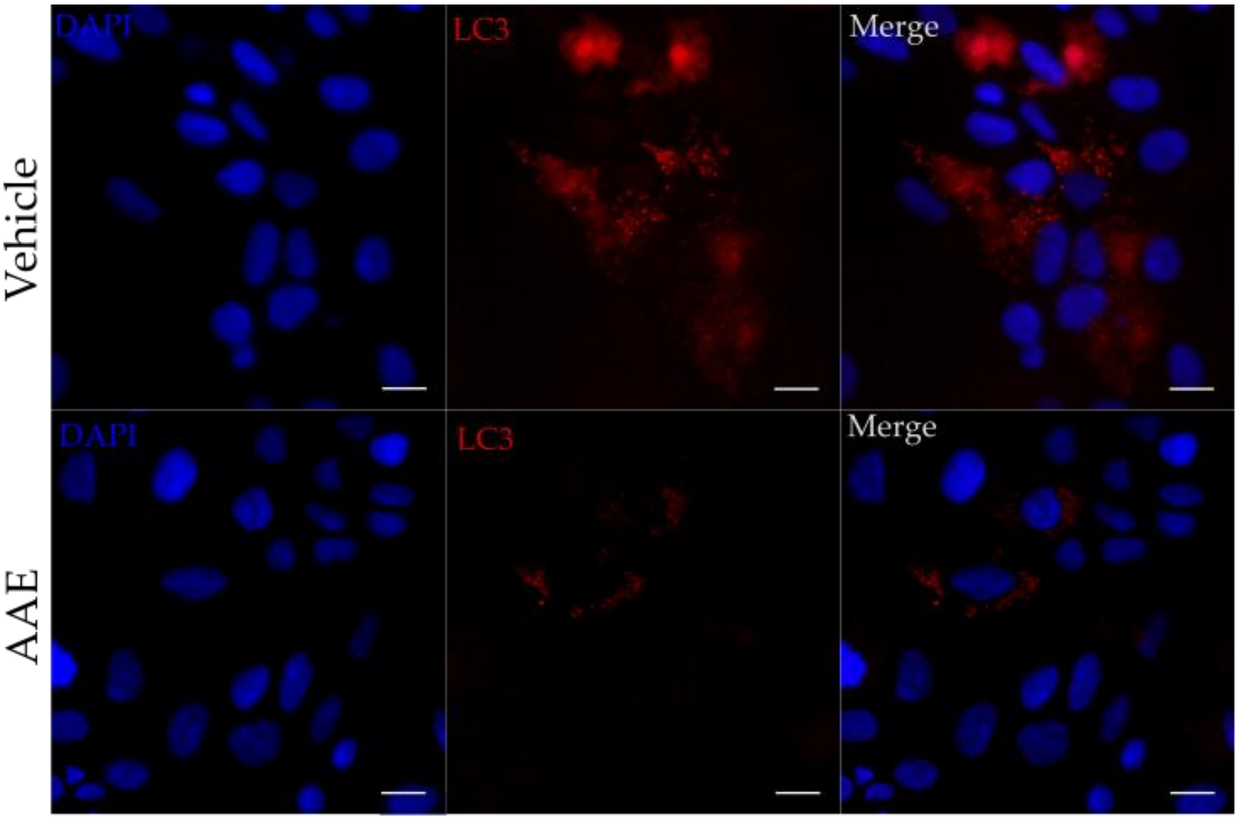

Figure S5. AAE does not induce autophagy in HuH7 cells. Non-confocal immunofluorescence showing LC3B positive punctuated structure in HuH7 cells grown in presence of AAE (400 mg/L) or the corresponding volume of vehicle (DMSO) for 72h. Cells were immuno-stained with an anti-LC3 antibody (red channel) to identify autophagosomes and with DAPI to reveal nuclei (blue channel). Scale bars correspond to 10 μm.

Table S1. Metabolites of HuH7 cells identified by DI-FT-ICR-MS and discussed in this manuscript.

Metabolite	m/z	Ionization	Error (ppm)
Glucose		$[M-H]^-$	-0.958
Lactate	113.02091	$[M+Na]^+$	-0.176
Maltose	341.1089	$[M-H]^-$	-0.894
Glucose 6-P	259.0222	$[M-H]^-$	-0.857
Proline	138.05254	$[M+Na]^+$	-0.936
Threonine	142.04746	$[M+Na]^+$	-0.801
Glutamine	169.05836	$[M+H]^-$	-0.845
Lysine	147.11280	$[M+H]^+$	-0.708
Histidine	156.07675	$[M+H]^+$	-0.980
Cysteine	120.01247	$[M-H]^-$	-0.774
Tryptophan	203.08260	$[M-H]^-$	-0.695
Taurine	124.00738	$[M+H]^-$	-0.948
Creatine	154.0586	$[M+Na]^+$	-0.890
Glutamic Acid	170.04237	$[M+Na]^+$	-0.931
Leucine	132.10190	$[M+H]^+$	-0.947
Tyrosine	180.06631	$[M+H]^-$	-0.703
Phenylalanine	188.06819	$[M+Na]^+$	-0.953
Aspartic Acid	132.03023	$[M-H]^-$	-0.918
α - GPC	257.23931	$[M-H]^-$	0.987
Ribose 5-P	229.01187	$[M-H]^-$	-0.905
Sedoheptulose	233.0631735	$[M+Na]^+$	-0.958
Sedoheptulose 7-P	289.0327	$[M-H]^-$	-0.934
NADP	663.1049	$[M-H]^-$	-0.832
Xanthine	153.04010	$[M+H]^+$	0.975
Adenosine	290.08597	$[M+Na]^+$	-0.981
Cytidine	282.04867	$[M+K]^+$	-0.957
Guanosine	322.05482	$[M+K]^+$	-0.956
Inosine	291.06999	$[M+Na]^+$	-0.962
Deoxy-Inosine	290.08570	$[M+K]^+$	0.968
GSH	306.07652	$[M-H]^-$	-0.783
Propionylcarnitine	218.13868	$[M+H]^+$	-0.983
Butyrylcarnitine	232.15433	$[M+H]^+$	-0.924
Valerylcarnitine	246.16998	$[M+H]^+$	-0.709
Citrate	191.0197	$[M-H]^-$	-0.974
Fumarate	115.00368	$[M-H]^-$	-0.801
Malate	133.01424	$[M-H]^-$	-0.878
